# A randomized clinical trial comparing early patient-reported pain after open anterior mesh repair *versus* totally extraperitoneal repair of inguinal hernia

**DOI:** 10.1093/bjs/znab354

**Published:** 2021-11-13

**Authors:** Markku Matikainen, Jaana Hellevi Vironen, Seppo Silvasti, Imre Ilves, Jyrki Kössi, Antti Kivivuori, Hannu Paajanen

**Affiliations:** 1 Department of Gastrointestinal Surgery, Kuopion Yliopistollinen Sairaala, Kuopio, Finland; 2 Department of Gastrointestinal Surgery, Helsinki University Central Hospital, Espoo, Finland; 3 Surgery, Pohjois-Karjalan Keskussairaala, Joensuu, Finland; 4 Surgery, Mikkeli Central Hospital, Mikkeli, Finland; 5 Surgery, Päijät-Hämeen Sosiaali- ja Terveysyhtymä, Lahti, Finland

## Abstract

**Background:**

This was a prospective, multicentre, non-blinded, randomized clinical trial involving two parallel groups of patients.

**Methods:**

Adult patients with symptomatic unilateral primary inguinal hernia were included in this study. Patients were enrolled and treated in five Finnish hospitals. Eligible patients were randomized by use of a computer-based program to receiving either open anterior repair (modified Lichtenstein) with glue mesh fixation or totally extraperitoneal (TEP) repair. The primary aims were to compare 30-day patient-reported pain scores and return to work after surgery between the two groups.

**Results:**

A total of 202 patients were randomized: 98 patients to TEP repair and 104 patients to open repair. All randomized patients received their allocated treatment. A total of 86 patients (88 per cent) in the TEP group and 94 patients (90 per cent) in the Lichtenstein group completed the 30-day follow-up. Patients experienced less early pain (*P* < 0.001) and used less analgesics after TEP repair, compared to those who had modified Lichtenstein repair. Two patients in the TEP group and five in the Lichtenstein group developed superficial wound infection (*P* = 0⋅446). Only one reoperation was performed in the Lichtenstein group due to haematoma.

**Conclusion:**

TEP inguinal hernia repair is associated with less early postoperative pain compared to the open glue mesh fixation technique.

**Trial registration:**

NCT03566433 (http://www.clinicaltrials.gov).

## Teaser

In this randomized clinical trial, we compared totally extraperitoneal (TEP) repair of groin hernia to open repair of groin hernia. The main outcome was patient-reported pain scores within the first 30 days after the index operation. Patients experienced less pain after TEP repair, compared to those who had open repair.

## Introduction

### Background

The lifetime risk of inguinal hernia surgery is estimated at twenty-seven per cent for men and three per cent for women^1^. Many different types of hernia repair have been described, without a consensus on the optimal surgical procedure^2^. A Lichtenstein repair^3^ is the most common open repair technique^2^. The Lichtenstein technique has evolved, with the modified techniques usually referred to as open anterior repair. Many guidelines consider the Lichtenstein procedure as a standard reference, because it is easy to learn and has short operating times and low costs, and it is possible to be performed under local anaesthesia for high-risk patients^2^^,^^4^^,^^5^. In addition to open repair, minimally invasive inguinal hernia repair has also gained popularity^2^.

The most frequently used laparoscopic techniques are transabdominal preperitoneal (TAPP) and totally extraperitoneal (TEP)^2^. TEP repair does not require intraperitoneal entry, and thus the incidence of intra-abdominal injuries is lower compared to that in TAPP repair^2^. In Nordic countries, the preferred laparoscopic technique is TEP repair^6^^,^^7^, with previous research reporting a decrease in early postoperative pain, with earlier return to work, when compared to the Lichtenstein method^4–7^. However, TEP repair is a more technically demanding operation and must be performed under general anaesthesia. Also, direct costs to the hospital are higher in TEP repair due to the need for general anaesthesia and disposable materials used^6^. Since widespread use of mesh repairs, recurrent hernias are rare^2^^,^^8^. However, chronic, as well as acute, pain remains a challenging problem^2^. Only very few studies have compared the Lichtenstein method *versus* TEP repair in terms of early (≤ 30 days) postoperative pain response^5^. Such data are important in young hernia patients who aim to resume physical activities and work as early as possible after the operation. Some studies have reported lower early and chronic pain when Lichtenstein repair is performed with glue fixation *versus* traditional suture fixation^9^. Therefore, this present study compared the patient-reported outcome measure (PROM) of early postoperative pain (≤ 30 days) after TEP repair without fixation and the Lichtenstein method with glue fixation of the mesh. The primary endpoint was early pain and recovery during the first 30 days following surgery. The secondary endpoints were return to work or normal physical activity and analgesic use.

## Methods

### Trial design

This was a prospective, non-blinded, randomized clinical trial. Patients were randomized in parallel groups, with an allocation ratio of 1 : 1. Two university hospitals and three central hospitals participated in this study.

The study was approved by the University of Eastern Finland Committee of Research Ethics and registered in ClinicalTrials.gov (ClinicalTrials.gov identifier: NCT03566433).

### Patients

Men and women aged from 18 to 80 years presenting with symptomatic unilateral primary inguinal hernia were included in this study. Patients with previous lower abdominal surgery (such as Pfannenstiel or lower midline laparotomy incision), high operative risk (ASA grade > III), bilateral hernia, large scrotal hernia, or femoral hernia were excluded. All operations were planned as day case. Participating surgeons recruited eligible patients consecutively from the waiting list, and written informed consent was obtained before randomization.

### Operative techniques

Six highly experienced senior hernia surgeons performed both open and TEP procedures. In the TEP technique, three ports were used: a 10-mm view port in the midline under the umbilicus and two 5-mm working ports under the 10-mm port. A balloon dissector was used to create the preperitoneal working space. Dissection was carried medially to the pubic bone, laterally to the anterior superior iliac spine, inferiorly under the spermatic cord, and for a few centimetres below the pubic bone. A polypropylene mesh (Optilene^™^, B. Braun, Melsungen, Germany) measuring 12 × 15 cm was used to cover the hernia orifice. The mesh was not secured with tackers or glue. In TEP repair, a volume of 4–5 ml of bupivacaine (Marcain® 5 mg/ml, AstraZeneca, Cambridge, UK) was locally injected in the skin wounds to anaesthetize trocar sites.

The open repair was performed as described by Lichtenstein^3^, with some modifications to the technique^10^. Tension-free hernioplasty was performed by using a 9 × 13-cm trimmed mesh (Optilene^™^). The sac of an indirect hernia was either resected or just inverted into the abdomen. If the hernia sac was large and direct, it was inverted with absorbable 2–0 sutures (Vicryl^™^ polyglactin, Johnson & Johnson Medical N.V., Machelen, Belgium). The mesh was placed between the conjoint tendon, the inguinal ligament, the pubic bone, and the internal oblique aponeurosis. The ilioinguinal, genitofemoral, and iliohypogastric nerves were identified, if possible, and carefully preserved. Mesh was fixed using *n*-butyl-2-cyanoacrylate tissue glue (Histoacryl®, B. Braun). Open repairs were always performed under local anaesthesia. The anaesthetic used for local infiltration was a 1 : 1 mixture of bupivacaine (Marcain® 5 mg/ml) and lidocain with adrenaline (10 mg/ml + 10 µg/ml, Orion, Kuopio, Finland). A bolus of 0.5–1.0 mg of intravenous alfentanil was administered (Rapifen®, AstraZeneca) if the patient felt pain during the operation.

No prophylactic antibiotics were used for either of the techniques. All patients were advised to return to normal daily activities and work as soon as possible after the operation. After surgery, patients were given 7 days of sick leave and those patients who needed more time were given an extension of their sick leave based on a telephone conversation. Ibuprofen or paracetamol were prescribed for postoperative pain.

### Outcomes

Demographic data, predisposing factors and hernia-related pain scores were recorded before the operation. Duration of the operation, the type and size of the hernia defect, length of hospital stay, and perioperative and immediate complications were recorded. PROMs were used to evaluate pain and recovery following surgery. Patients were given a pain diary on their discharge from hospital. Patients reported postoperative pain at rest, during exercise, and on coughing. Pain level was recorded twice a day for 7 days after surgery and then postoperatively at days 14, 21, and 30. A numerical rating scale (NRS) of 0 to 10 was used. Patients reported the day of complete recovery from the operation when they could perform normal physical activities or work without difficulty. Use and doses of all analgesics were recorded. Computerized patient records in each hospital for every patient were screened 1 month after surgery for further hospital visits, complications, and reoperations.

### Sample size

Power calculation showed that 80 subjects per treatment group were needed for the study to achieve a statistical power of 0.90, with a *P*-value of 0⋅05 (two-tailed). Calculations were made for postoperative pain at 7 days after surgery, assuming a pain difference of twenty per cent between treatment groups. Allowing a dropout rate of twenty per cent during 30-day follow-up, the study was set up to include randomization of > 200 patients.

### Randomization

**Fig. 1:**
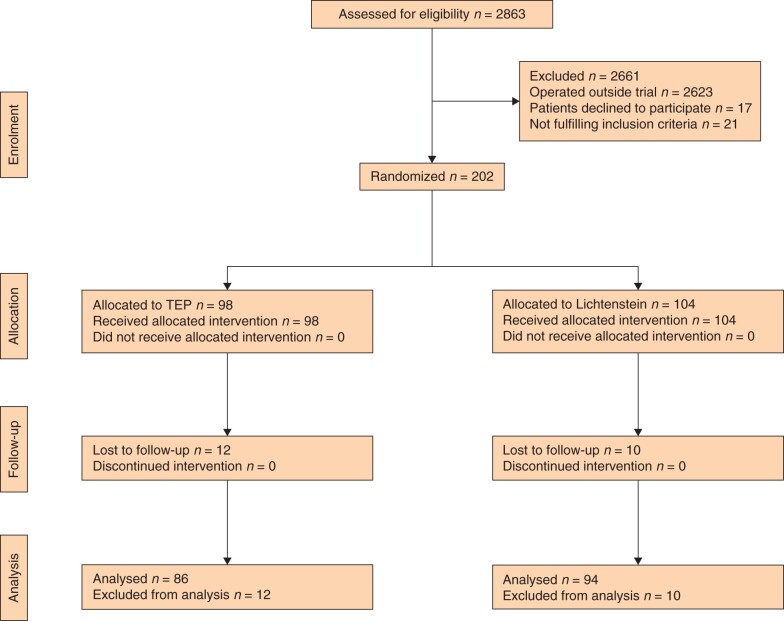
Study flow chart

Immediately after giving informed consent, patients were randomized by participating surgeons at each centre to having either Lichtenstein or TEP surgery (*Fig. 1*). Block randomization in blocks of 40 patients in each centre was performed using a web-based HUSeCRF (University of Helsinki, electronic case report form) program.

### Statistical analysis

Data analysis was carried out with SPSS for Windows, Release 27.0 (IBM SPSS, Chicago, Illinois, USA). Continuous data were presented as means, and analysed with Student’s *t* test (two-tailed) for parametric data and with Mann–Whitney U test for non-parametric data. Categorical variables were analysed using Pearson’s chi-squared test and Fisher’s exact test. A mixed model analysis was used to analyse pain scores. *P* < 0⋅05 was considered as significant for all tests.

## Results

A total of 202 patients who underwent inguinal hernia surgery between June 2017 and October 2020 consented to participate in the study. Baseline patient data are provided in *Table 1*. Ninety-eight patients were randomly allocated to the TEP group and 104 to the Lichtenstein group (*Fig. 1*). A total of 86 patients (eighty-eight per cent) in the TEP group and 94 patients (ninety per cent) in the Lichtenstein group completed follow-up by completing and returning their pain diaries.

**Table 1:** Patient and operation characteristics

	Lichtenstein (*n* = 104)	TEP (*n* = 98)	***P*-value**
**Age (years)** *	56 (22–77)	53 (18–79)	0.17
**Sex ratio (M : F)**	95:05:00	96:05:00	0.803
**BMI (kg/m^2^)** *	25	25	0.967
**Smoker**	18	14	0.827
**Duration of symptoms (months)** *	3	3	0.876
**Combined hernia**	2	4	
**Preoperative pain** * †	3	3	0.19
**Preoperative need of analgesics for hernia pain**			0.68
Not needed	74	76	
1–4 times per month	17	19	
1–6 times per week	7	3	
Daily	2	2	
**Absence from work due to hernia**	11	7	0.424
**Outpatient surgery**	99	96	0.209
Operating time (min)*	37	36	0.322
Hernia orifice size (cm)*	2	2	0.223
**Type of hernia**			0.578
Lateral hernia	70	65	
Medial hernia	28	31	

Values in parentheses are percentages unless indicated otherwise.

*Values are median (i.q.r.).

†Number rating Scale (0–10).

### Operations

Every patient received their allocated treatment. Mean operating times were similar: TEP 36 minutes (range 17–80) *versus* Lichtenstein 37 minutes (range 20–85) (*P* = 0⋅318). No intraoperative complications or conversions from laparoscopic to open surgery were observed. One patient in the Lichtenstein group (monitoring due to sleep apnoea) and four patients in the TEP group (two patients due to urinary retention and two for social reasons) needed overnight hospital admission. Few, and mostly minor, postoperative complications were reported. One patient in the Lichtenstein group needed reoperation due to haematoma, whereas no reoperations were performed in the TEP group. Five patients in the Lichtenstein group *versus* two patients in the TEP group needed antibiotics for minor surgical site infection (*P* = 0⋅446). No recurrent hernias were observed during the 1-month follow-up.

### Primary and secondary outcome

**Fig. 2:**
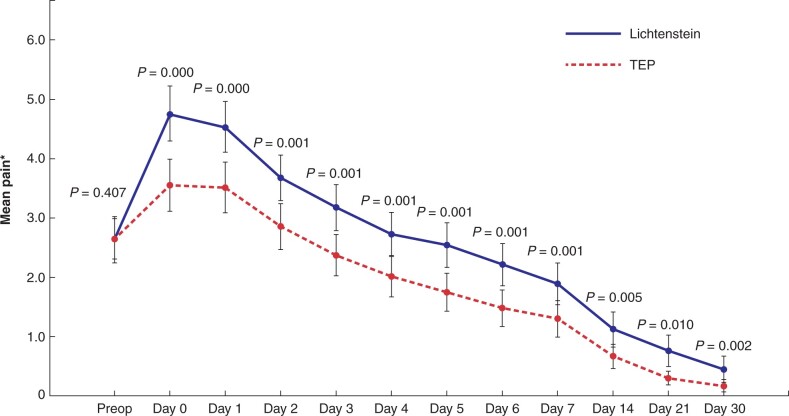
Postoperative pain scores *Numerical rating scale. Error bars: 95 per cent confidence interval. *P*-values analysed with independent samples *t* test.

**Fig. 3:**
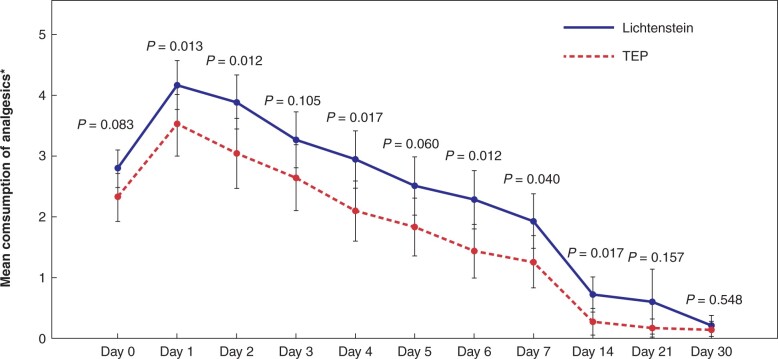
Consumption of analgesics. *One unit is equivalent to 1 gram of paracetamol or 600 mg of ibuprofen per orally *One unit is equivalent to 1 g oral paracetamol or 600  mg oral ibuprofen. Error bars: 95 per cent confidence interval. *P*-values analysed with independent samples *t* test.

Postoperative pain scores were lower after TEP repair, compared to Lichtenstein repair (*P* < 0⋅001), at all postoperative measurement points (*Fig. 2*). Patients in the TEP group returned to work or normal physical activities slightly earlier than those in the Lichtenstein group: 14 days (range 1–31) *versus* 17 days (range 1–84), respectively (*P* = 0⋅048). However, on exclusion from analysis of one patient from the Lichtenstein group who had an exceptionally long recovery time (84 days), then there was no statistical difference in recovery times between the two groups. Use of analgesics was more frequent in the Lichtenstein group, although this difference levelled off towards the end of the 30-day follow-up, with significant differences only observed at days 1, 2, 4, 6, 7, and 14 (*Fig. 3*).

## Discussion

As inguinal hernia repair is one of the commonest surgeries performed worldwide, it is important to find the optimal treatment^2^. As open Lichtenstein repair is less expensive and easier to teach and learn, it is still commonly used in many patients who could potentially benefit from minimally invasive surgery. However, the current trend is to offer patients tailored hernia surgery. Especially in young patients who may appreciate a rapid return to their usual activities, it is important to know whether endoscopic repair has advantages over the open technique. In the present study, TEP repair resulted in less painful recovery, compared to the open Lichtenstein method. The clinical significance of this finding was emphasized by the observation that TEP patients required less analgesics than patients who received Lichtenstein repair.

The absolute difference in NRS values between the two groups was not large. However, we consider the difference to be clinically significant, as in the TEP group, the mean NRS value decreased to the mild pain level within 2 days, whereas in the Lichtenstein group, the corresponding pain level was reached at day 4.

In all previously published studies, the open surgical technique included mesh fixation with a penetrating method. We aimed to minimize surgical bias by using non-penetrating fixation in the open group and the same mesh in both groups. Performing TEP repair without fixation or open repair with glue fixation has been previously evaluated to be safe and possibly less painful, compared to penetrating fixation^9^^,^^11–13^. Our present study findings are in keeping with these studies comparing recovery after TEP repair *versus* after Lichtenstein repair with penetrating mesh fixation^4^^,^^6^^,^^7^^,^^14^. In a well planned study, Eklund and co-workers^7^ compared the TEP *versus* the Lichtenstein method by carefully evaluating postoperative pain once per day for the first week and then once per week up to 6 weeks. Their results are in agreement with our findings, demonstrating faster and less painful recovery after TEP repair. The present study also showed that even with use of non-penetrating fixation in open Lichtenstein repair, TEP repair was superior in terms of early recovery.

In many previous studies, the operating time for TEP repair was shown to be significantly longer^5^. In the present study, the operating time in both groups was short, with no difference between the two groups which may reflect the experience in, and skill for, both techniques by the operating surgeons participating in this study. This highlights the fact that the operating time itself is not a relevant factor when choosing the preferred operating technique.

The obvious limitation in our study is the short follow-up period, with few patients still reporting pain 4 weeks after surgery – even early recurrences are usually diagnosed later than 30 days after surgery. Also, there was no clinical or monitoring, possibly resulting in some surgical complications or early recurrent hernias being undetected. The strength of our study was its carefully planned, multicentre design, with robust randomization, standardized surgical techniques with few complications, and successful completion and return of patient pain diaries to minimize potential biases. Also highly experienced senior surgeons performed all operations, which helped to minimize technical bias in terms of causing excess pain. When interpreting our results in the context of clinical practice, it is important to consider that due to the longer learning curve, many studies have demonstrated inferior results when TEP repair is not performed by experienced surgeons^15^.

## Funding

No funding was received.

*Disclosure.* The authors declare no conflict of interest.

## Supplementary Material

znab354_Supplementary_Data
